# Designing interdisciplinary education in digital public health: a position paper

**DOI:** 10.3389/fpubh.2026.1866630

**Published:** 2026-06-30

**Authors:** Conrad Attard, Anabelle Macedo Silva, Syed Ahmed, Brian Li Han Wong, Roberta Sultana, Emiliano Albanese, Ermira Tartari

**Affiliations:** 1 Faculty of Information and Communication Technology, University of Malta, Msida, Malta; 2 Instituto de Estudos em Saúde Coletiva/IESC (Public/Collective Health Institute), Federal University of Rio de Janeiro/UFRJ, Rio de Janeiro, Brazil; 3 Leibniz Science Campus on Digital Public Health/LSCDiPH, BIPS, Bremen, Germany; 4 Business Research Unit Digital Health International, ISCTE–University Institute of Lisbon, Lisbon, Portugal; 5 Department of International Health, Care, and Public Health Research Institute, Maastricht University, Maastricht, Netherlands; 6 Digital Public Health Task Force, Association of Schools of Public Health in the European Region (ASPHER), Brussels, Belgium; 7 Faculty of Biomedical Sciences, Institute of Public Health, Università della Svizzera Italiana, Lugano, Switzerland; 8 Faculty of Health Sciences, University of Malta, Msida, Malta

**Keywords:** digital health literacy, digital public health, ethics and regulation in digital health, health workforce competencies, interdisciplinary education

## Abstract

The rapid digital transformation of health systems worldwide demands an interdisciplinary public health workforce proficient in digital technologies, ethical frameworks and systems-level thinking. However, current public health curricula remain largely discipline-specific and fail to systematically embed digital competencies or foster collaboration across sectors such as technology, ethics, law, and policy. This position paper emerges from a high-level workshop held during the Digital Public Health Conference 2025, where international experts and educators debated strategies for advancing interdisciplinary education. Drawing on multilevel evidence, including participant reflections, international frameworks, and case studies, we identify and analyse five critical challenges shaping the future of digital public health education: (1) embedding ethics and regulation into digital public health education; (2) strengthening digital public health literacy with a salutogenic approach; (3) defining interdisciplinary competencies for the future workforce; (4) integrating real-world applications into classroom learning; and (5) enabling long-term, cross-border collaboration in education. For each theme, we propose actionable goals rooted in pedagogical innovation, policy alignment, and institutional governance. Designing inclusive, ethical, and practice-informed education models is essential to prepare a digitally fluent and socially accountable public health workforce.

## 1 Introduction

The increasing complexity of global health challenges, driven by rapid technological advancements, necessitates a public health workforce equipped with interdisciplinary competencies. In this context, we refer to professionals engaged in population-level functions, such as health promotion, disease prevention, surveillance, and policy rather than the broader clinical healthcare workforce. The public health workforce is uniquely and disproportionately impacted by the digitization and digitalization of data systems, population monitoring, and decision-making processes. Emerging domains such as artificial intelligence, precision public health, digital epidemiology, and serious games are reshaping health promotion, disease prevention, and public health policy (1). However, most public health education programmes do not yet systematically incorporate the teaching and learning approaches required to develop digital competencies and skills among graduates. Addressing this gap requires not only curriculum reform but also a rethinking of strategies that foster collaboration across disciplines, including public health, data science, information and communication technology, law, and ethics.

This position paper draws on a panel debate held at the Digital Public Health Conference 2025, where international experts examined key priorities in designing interdisciplinary education for digital public health. The discussion focused on five interrelated areas: the integration of ethics and regulation into digital public health curricula, health literacy in digital contexts, interdisciplinary competency frameworks for the future workforce; experiential and practice-based learning approaches, and sustainable cross-border academic collaboration. Recent initiatives reflect this emerging context, including the European Health Data Space (EHDS, 2025), the EU’s most ambitious digital health regulatory reform to date (2). The XiA (Erasmus+, 2024–2028) (3) and SUSA projects (4) are building workforce competencies and lifelong learning pathways across institutional and national boundaries, and models such as WHO Collaborating Centres (5) and European University Alliances (e.g., EPICUR, CIVIS) (6, 7) demonstrate that sustained, institutionally embedded cross-border education is achievable at scale. Building on these themes, this paper examines key challenges and proposes directions for strengthening interdisciplinary education in digital public health.

## 2 Methods

This paper reports on a 90-min panel discussion held at the 10th International Public Health Conference in Madeira/Portugal, July 2025, convened to advance interdisciplinary digital public health education. Participants represented diverse relevant disciplines and pursued three objectives: (1) identifying potential opportunities and challenges of interdisciplinary education in public health programmes; (2) sharing case studies on digital health literacy within public health education; and (3) co-designing strategies for fostering interdisciplinary learning at programme and course levels. Drawing on these reflections, international frameworks, and case studies, five critical challenges were identified and examined against extant literature. All participants provided written informed consent and participation was voluntary.

## 3 Workshop insights and evidence-based perspectives

### 3.1 Challenge 1: embedding ethics and regulation into digital public health education

Despite the accelerating integration of digital technologies in healthcare, digital public health education remains fragmented, leaving professionals ill-equipped to navigate the associated ethical, legal, and regulatory complexities. In particular, ethics, health equity, and data protection principles are not yet systematically embedded in many curricula. Evidence highlights a lack of interdisciplinary educational approaches that integrate public health competencies with fundamental rights, health and digital equity, regulatory data protection perspectives. Proposed solutions include integrated curricula embedding these dimensions in a coherent and applied manner.

Notwithstanding, current curricula often fail to integrate ethical, equity, and regulatory reflections into the practical use of digital tools. As a results, technologies introduced without adequate ethical and regulatory guidance, sufficient investment, or appropriate workforce preparation may undermine core public health values such as equity, accessibility, and safety. They may also compromise data protection and other fundamental rights (8), exacerbate social inequalities, widen the digital divide (9) and raise concerns related to digital sovereignty (10–12). In this context, the digital divide should not be understood only at the individual level, where patients may face barriers related to digital literacy or access to technology, but also at collective and institutional levels (13). These include limitations among health professionals, and health systems in adopting and implementing digital tools that adequately respond to population needs and vulnerabilities.

Furthermore, while patients have the fundamental right to receive care, populations also have a collective interest in protection from public health threats, particularly during infectious disease outbreaks. These tensions illustrate the need to balance individual and collective dimensions of fundamental rights proportionately. They also highlight the importance to distinguish between the fundamental right to health and its expression through policy-dependent rights of access to healthcare services.

Ethical and regulatory complexities further complicate this landscape. Contemporary rights-based discourses, while rooted in democratic ideals, risk being co-opted or diluted in the face of state-led decisions during health emergencies. These dilemmas underscore the urgent need for a renewed societal dialogue on the challenges posed by digital transformation and personal data massive collection, one that reconsiders the digital dimensions of fundamental rights (14) and health data governance (15) underpinning healthcare delivery in the digital age, as well as assessing public health systems maturity regarding digital public health (16), and its challenges in order to prepare public health workforce, with the capabilities needed in the emerging context of digital technologies, AI and health data use.

In response, the panel proposed several educational strategies. First, research, education and training programmes should explore how digital technologies can strengthen ethical foundations- such as equity justice and data protection (17). This requires moving beyond teaching ethics as a theoretical add-on and instead embedding it within practical, technology-oriented scenarios. Second, curricula should promote structured interdisciplinary engagement with human rights, public health ethics, and data protection law, enabling students to understand and navigate trade-offs between competing rights and interests (18, 19). Third, educational programmes should address the implications of health equity, the right to health, and regulatory obligations for the design and implementation of digital public health interventions. There is a need to examine how digital transformation redistributes risks, costs, and benefits across society. Transparent governance and participatory decision-making are important components of a fair digital public health ecosystem (20). A broader ethical and regulatory lens, incorporating dimensions such as equity in health, determinants of health, human fundamental rights, care and cure, was proposed as a foundation for addressing systemic injustices, occasionally fostering fairer new social developments. Embedding such approaches in education would better prepare future health professionals to lead digital transformation in ways that are technically sound, ethically grounded and socially accountable. Digital public health education should integrate ethics, data protection, health equity, digital inclusion, and fundamental rights into curricula through learner-appropriate and practice-oriented teaching approaches aligned with evolving policy and regulatory frameworks (21, 22).

### 3.2 Challenge 2: strengthening digital public health literacy with a salutogenic approach

Digital public health interventions may contribute to improve health outcomes in populations by enhancing self-efficacy, empowerment and access to care. However, the current focus on healthcare (mainly disease management) over health promotion and prevention, compounded by limited integration of digital public health literacy, limits the scope and potential benefits of the digital transformation in public health. Evidence from WHO/OECD frameworks, salutogenic models, and implementation studies supports a broader approach that prioritises empowerment, co-creation and equity in digital public health.

Health literacy is increasingly recognised as a key determinant of health equity and digital inclusion (23). Within digital public health, however, it is often narrowly defined as the ability to access and understand digital information, overlooking deeper competencies such as empowerment, self-efficacy, and critical evaluation of digitalized sources (24, 25). Addressing this limitation requires a shift from a pathogenic model, centred on illness, risk, and deficit, to a salutogenic model that promotes resilience, self-management, and sustained healthy living through digital tools (26). This reflects a broader shift from healthcare to public health thinking, from treating disease to enabling well-being.

Portugal’s national smoking cessation programme illustrates the gap between public health need and digital support capacity: despite high smoking prevalence, smoking cessation services remain under-resourced, with limited access to evidence-based interventions. Upstream modulators are poorly addressed, and with very modest leverage of digitalization. In the United States, although smoking cessation websites are widely used, their scientific quality and effectiveness vary considerably, and is communicated unidirectionally, often in patronizing forms. Together, these cases highlight the need for a more salutogenic approach to digital public health, based on co-created interventions that promote empowerment, resilience, and equitable well-being (SDG 3) (27). The active involvement of members of the target population and relevant stakeholders is indispensable to design and implement need- and person-centered digital interventions that are not mediated by human facilitators.

The cultivation of self-management capabilities, especially in chronic conditions such as tobacco dependency and pulmonary disease is critical. And yet, standard healthcare professionals are poorly trained, and possess limited competencies and skills in both communication and empowerment techniques. Public health education can effectively leverage digital transformation to can address intersecting issues including: diagnostic delays through better digital infrastructure, promoting interoperability in health information systems, and developing quality indicators to support patient decision-making, and digitally monitored self-care/ management. Attending to predictors of motivation and an individual’s sense of coherence (SOC)—the extent to which one perceives life as comprehensible, manageable, and meaningful—was identified as critical to long-term success in health behaviour change. So called m- and e-health approaches can facilitate access and use of services and interventions, and improve their sustainability, while reducing cultural and contextual barriers.

At the same time, the digital transformation profoundly modifies the digital environment, or “digital lifeworld,” as discussed, which now represents a core societal structure. Exclusion from this domain risks becoming a form of structural health inequality, or a “Generalised Resistance Resource Deficit” (GRR-D), impeding one’s ability to cope, adapt, and recover, and to actively participate in social life in meaningful manners. Therefore, embedding digital public health within health literacy education must not only equip citizens with technical skills but also with socio-ethical awareness, cultural sensitivity, and the ability to foster digital empowerment. In healthcare, telehealth services, health applications, and electronic health records have been reported as predominant intervention types, highlighting the need for standardised frameworks to enhance interdisciplinary collaboration, evaluation, and scalability (28). But adoption and adherence to, and compliance with digitally transformed healthcare tools and contexts can not only hinder the expected health benefits and improved efficiency and cost-effectiveness of care provision, but also constitute a barrier that can exacerbates health and care inequalities (see below). Complementing this, a comprehensive 182-question framework spanning 12 domains has been developed to ensure systematic consideration of ethical, legal, and sociocultural factors in DiPH intervention design (29). Such a curriculum would enable future public health professionals to interoperate effectively with healthcare professionals and, most importantly, a broad range of stakeholders, to innovate responsibly and equitably across diverse populations and systems.

Fragmented systems, poor interoperability, and infrastructure deficits undermine DiPH integration, compounded by nontechnical barriers including ethical concerns, policy gaps, and resource constraints that disproportionately burden vulnerable populations and exacerbate digital divides (30–32). Digital literacy gaps further entrench health inequities, yet evidence demonstrates that targeted interventions, including; co-designed educational modules, online training programmes, and whole-population campaigns, effectively improve eHealth tool usage and health outcomes among older persons and low-income populations (33, 34). These interconnected barriers reinforce the urgent need for scalable public education, participatory design frameworks, and strategic infrastructure investments to advance equitable DiPH implementation. Digital public health competencies and skills will enable the future public health workforce to bridge across highly diverse and complementary disciplines and specialists, while ensuring a solid grounding in the foundational public health principles including equity and justice. This bridging capacity requires interdisciplinary competencies.

### 3.3 Challenge 3: defining interdisciplinary competencies for a future workforce

Addressing the lack of shared skill sets across information and communication technology, health, law, and policy remains a key challenge, yet interdisciplinary education offers the benefit of producing digitally fluent and adaptable graduates. Existing competency frameworks, such as those from ASPHER and AMIA provide evidence for a proposed core competency matrix, and industry co-designed curricula to bridge these divides.

This challenge has become more urgent considering the European Health Data Space (EHDS), which will require professionals across public health, clinical, technical, and policy settings to engage with shared data standards, interoperable infrastructures, and new governance mechanisms (35, 36). For education, the significance of the EHDS lies less in its regulatory detail than in its workforce implications: professionals will need not only legal and ethical awareness, but also a practical understanding of data models, interoperability standards, and data-access pathways. The digital transformation of health systems and the implementation of the European Health Data Space (EHDS) requires a public health workforce capable of operating at the interface between health, technology, and policy (11, 37, 38).

The EHDS represents the most ambitious regulatory and infrastructural reform of digital health in the European Union to date, designed to establish a unified ecosystem of rules, standards, digital infrastructures, and governance mechanisms for the use of health data across Member States (2). Building on two decades of voluntary cross-border collaborations and EU-funded initiatives, and on the existing MyHealth@EU infrastructure for primary data exchange, the EHDS now provides a legally binding framework that supports both primary use of health data—ensuring citizens’ seamless access to their electronic health records and continuity of care across borders—and secondary use, enabling researchers, public authorities, and innovators to securely access de-identified datasets for purposes such as surveillance, policy-making, and public health research (39–41). Central to this transformation is the introduction of a harmonised European Electronic Health Record Exchange Format (EEHRxF), first recommended in 2019 (42) and now a key provision of the EHDS, and the requirement that EHR systems incorporate interoperable, machine-readable components by defined implementation deadlines between 2028 and 2034 (43). The regulation also establishes national Health Data Access Bodies and a secure data-access architecture to govern secondary-use requests (44, 45).

Taken together, these reforms will significantly elevate expectations on the healthcare workforce readiness. Public health professionals will interact in unprecedentedly complex and varied ways with other healthcare professionals across clinical and technical domains, amongst others, and they will need to understand interoperability standards, governance requirements, and data access pathways to fully leverage the potential of the EHDS, within and beyond the operational and scope boundaries of health services. There are good examples of innovative projects in the field of interoperability within health services and across healthcare, including the European Commission co-funded an international project with its Erasmus+ instrument; the Xpanding Innovative Alliance (XiA) project is a 4 years and 4 million initiative aiming at bridging this gap comprehensively across the health workforce and competences required to permit the implementation of the EHDS and the EEHRxF.^1^ Importantly, XiA collaborates formally with the ASPHER group and the workshop held during the Digital Public Health Conference 2025 marked the practical start of their joint effort to prepare public health professionals for the evolving European digital health reality that is unfolding now. This workforce is expected to operate also beyond healthcare services, and to guarantee seamless operability across and between the WHO 12 Essential Public Health Functions (EPHFs), that imply cross-disciplinary and inter-sectoral collaboration. Table 1 encompasses the core challenges and proposed approaches discussed in Challenge 3.

**Table 1:** Proposed challenges and approaches pertaining to Challenge 3.

Theme	Core challenge	Proposed approach
Fragmented & uneven competencies	Public health spans data analytics, policy design, epidemiology, and community engagement—making a single standardised competency framework neither feasible nor appropriate.	Differentiated learning pathways aligned with distinct professional subgroups within the public health field.
From awareness to specialisation	Most professionals know GDPR but few understand EHDS architecture or interoperability standards (HL7 FHIR, SNOMED CT, ICD, openEHR). Two gaps persist: limited awareness and insufficient technical literacy.	Foundational education must cover technical terminology, data models, and system integration protocols before enabling role-specific specialisation.
Tailoring learning pathways	One-size training fails diverse roles. Policy professionals need data governance skills; data analysts need interoperability, data quality, and algorithmic bias training.	A core competency matrix defining transversal and specialised skills, underpinning modular curricula and micro-credential pathways for tailored portfolio building.
Pedagogical innovation & lifelong learning	Generational and institutional disparities in digital competence exist. Early-career professionals are often digitally fluent; large segments of the existing workforce are not.	Micro-content learning blocks (MCLBs)—short, focused units embedded in workplace or academic programmes—enabling flexible, self-paced continuous professional development.
Collaborative curriculum co-design	Educational content risks falling behind evolving standards, regulatory requirements, and labour-market needs without sustained cross-sector collaboration.	Joint degree programmes and co-designed curricula with technology, industry, professional associations, and government agencies; reinforces both technical and organisational interoperability.
Future-ready workforce	Preparing the public health workforce for the digital era requires more than generic digital literacy—it demands recognition of role diversity and field interdisciplinarity.	A stratified, competency-based model producing graduates who are digitally fluent, adaptable, ethically aware, and capable of advancing the EHDS vision of integrated, data-driven health systems.
Future directions (XiA–ASPHER)	Workshop insights need to move from theoretical frameworks to practical implementation tools that map real educational needs across professional roles.	Development of a “Public Health Persona Typology” and an “Awareness Starter Pack”—curated micro-content learning blocks providing jargon-free orientation on the EHDS, EEHRxF, and interoperability standards.

HL7 FHIR, Health Level Seven Fast Healthcare Interoperability Resources; SNOMED CT, Systematized Nomenclature of Medicine—Clinical Terms; ICD, International Classification of Diseases; openEHR, Open Electronic Health Record; EHDS, European Health Data Space; EEHRxF, European Electronic Health Record Exchange Format.

Preparing the public health workforce for the digital era requires more than generic digital literacy. It calls for a stratified, competency-based approach that recognises the diversity of professional roles and the interdisciplinary nature of the field (46). Moving from fragmented training towards a coherent and collaborative model of competency development could help produce professionals who are digitally capable, adaptable, ethically informed, and better equipped to contribute to integrated, data-driven health systems. As a practical next step, insights from the workshop will inform the development of a public health persona typology to map educational needs across roles such as policy, data science, education, and field practice, alongside an introductory set of micro-content learning blocks designed to provide accessible orientation to the EHDS, the EEHRxF, and key interoperability standards.

### 3.4 Challenge 4: integrating real-world applications into classroom learning

Over-reliance on lecture-based formats remains a key challenge in public health workshop delivery, limiting participant engagement and practical skill development. Evidence from project-based learning research and EU institutional best practices demonstrates that active pedagogies significantly enhance engagement, deep learning, and employability outcomes. To address this, proposed solutions including hackathons, capstone projects, digital simulations, and virtual reality provide hands-on, immersive learning experiences that better equip participants with the competencies needed to navigate complex digital health environments. And yet, traditional and ‘analogic’ pedagogical methods can be integrated into innovative ones to stimulate critical thinking and human-machine-human interaction.

Traditional lecture-based approaches in public health education are increasingly misaligned with the demands of a rapidly evolving digital and interdisciplinary health landscape (47). Mental health, for example, requires teaching formats that reflect the complexity of real-world systems, stakeholder dynamics, and digital transformations that are re-shaping the world (48, 49). The workshop discussion emphasised that experiential, project-based learning is essential for preparing learners to work across sectors and to engage with the technological, social, and governance challenges that characterise modern public health practice.

Interdisciplinary collaboration is not merely a pedagogical technique but also a professional competence that must be intentionally developed. Meaningful collaboration is often hindered by competing institutional priorities, disciplinary silos, and differing professional cultures. Educational settings should therefore create opportunities for learners to build trust, communicate across disciplinary boundaries, and engage in shared problem-solving. Action-learning approaches were identified as especially valuable in this regard, as they enable students to work collectively on complex challenges while developing communication, reflexivity, and emotional intelligence alongside technical expertise (50, 51).

There is a need to shift from isolated teaching of digital skills toward the cultivation of “collaboration readiness.” This includes competencies such as leadership, project management, conflict resolution, and ethical communication. Rather than a focus solely on static skills, curricula must prepare students for dynamic contexts where roles, technologies, and priorities evolve. Similarly, it has been argued that health professions education must go beyond isolated competency development to produce collaborative-ready professionals equipped to work within complex health systems (52). In the context of mental health, where service provision frequently intersects with law, education, and community structures, interdisciplinary team-based learning becomes a mirror of actual practice.

The development of a “partnership readiness assessment” as a tool for students to reflect on their own assumptions, biases, and collaborative competencies before engaging in team-based activities was highlighted. This tool supports a project-based learning (PBL) and design thinking in EU academic programs, which shows that experiential, team-based education fosters not only deeper learning but also long-term employability and innovation capacity (53). Recommendations include the adoption of interdisciplinary capstone projects, health-focused hackathons, and digital simulations or VR-based role-play environments. These real-world applications foster cognitive flexibility, systems thinking, and empathy, skills that are particularly vital in digital mental health contexts, where interventions must balance technological capability with human dignity and psychosocial support.

Alongside self-reflective tools, structural accountability mechanisms are necessary to prevent the diffusion of responsibility that characterises large group projects. A RACI framework, clarifying who is Responsible, Accountable, Consulted, and Informed for each project task provides an effective scaffold for interdisciplinary teams (54). Embedding RACI or equivalent role-assignment tools early in collaborative modules teaches students not only how to complete a task, but how to govern shared work. This mirrors real-world public health practice, where multi-agency initiatives succeed or fail based on the clarity of role boundaries, not only on technical competence. Curricula should therefore include explicit instruction in collaborative governance, including role negotiation, conflict resolution protocols, and milestone accountability, as transferable professional competencies.

Bringing real-world applications into the classroom is essential for preparing future professionals to work effectively in digitally mediated and interdisciplinary public health environments. Embedding experiential learning within curricula can help cultivate the adaptability, ethical awareness, and collaborative leadership needed to support responsible innovation in digital public health (55, 56).

### 3.5 Challenge 5: enabling long-term, cross-border collaboration in education

Short funding cycles, fragmented governance, and cultural barriers present persistent challenges to sustaining international collaboration in digital public health education. Evidence from established frameworks such as Erasmus+, COST, and Horizon Europe, alongside WHO-UNESCO guidance, demonstrates that structured international partnerships yield significant benefits including cross-cultural competency development and global knowledge sharing. Proposed solutions include virtual collaborative networks, joint interdisciplinary modules, and institutional governance frameworks designed to overcome structural and cultural barriers, ensuring continuity and equity in international digital health education partnerships.

Achieving durable and effective interdisciplinary education requires more than pedagogical innovation alone. It also depends on robust governance structures, sustained financing, and intelligent use of digital platforms (11, 57). The central challenge lies in moving beyond short-term academic partnerships towards institutionalised, international collaborations capable of withstanding funding disruptions, leadership turnover, and geopolitical uncertainty.

Formalised governance models that promote accountability, transparency and shared ownership are critical. European University Alliances, such as EPICUR and CIVIS, demonstrate how joint strategic planning can be sustained through shared leadership and dedicated coordination structures, while WHO Collaborating Centres operate through defined terms of reference, structured workplans, and regular reporting mechanisms. Such models foster continuity by embedding collaboration within institutional structures rather than relying on individual projects or personal networks.

Realising this in practice requires that any cross-border educational initiative designates accountable leadership from the outset, including a steering committee with institutional mandates, a coordinating body with operational continuity, and defined reporting lines to funding bodies and partner institutions. The WHO Collaborating Centres model is instructive: formal designation and biennial workplan reviews ensure continuity independent of individual champions (5). Equivalent formalisation within Erasmus+ consortia and European University Alliances would reduce initiative mortality when funding cycles close or leadership changes. A distinction must also be drawn between the roles of international and national bodies. International organisations, including WHO (58), the European Commission, and ASPHER, set normative frameworks, interoperability standards, and funding instruments, while national and regional authorities are responsible for contextualising these within local health systems and regulatory environments. Without this articulation, international frameworks risk remaining aspirational. Curricula must therefore equip graduates to navigate governance across levels, understanding where standards travel, where adaptation occurs, and where accountability lies.

Sustainable international collaboration requires a diversified financing ecosystem that does not depend on any single source. Public funding through EU instruments, including Horizon Europe for research-education integration, Erasmus+ for curriculum mobility, and structural funds for infrastructure, provides a foundation, but must be complemented by institutional budget commitments from partner universities and health systems to ensure continuity beyond grant cycles. Public-private partnerships, such as the Innovative Medicines Initiative (59), offer co-investment models that align industry and public health priorities, while crowdfunding platforms and philanthropic foundations can mobilise targeted supplementary resources. Critically, financial planning must be embedded in governance structures from inception, not treated as an afterthought, with multi-year budget frameworks, contingency reserves, and defined cost-sharing arrangements between partners established at the outset.

Digital infrastructure represents a strategic enabler of cross-border collaboration. Learning management systems with analytic capabilities, collaborative digital platforms, and immersive virtual learning environments can enhance both teaching and institutional coordination across countries and time zones (60–62). Beyond course delivery, such technologies reduce the transactional burden of international collaboration and improve organisational agility.

Governance must nonetheless precede innovation. Without clear mechanisms for decision-making, conflict resolution, and resource distribution, even the most promising interdisciplinary initiatives are unlikely to scale or be sustained. Long-term collaboration in digital public health education therefore requires not only innovative teaching models, but also governance literacy, institutional commitment, and active engagement with funders, policymakers, and external partners.

**Figure 1:**
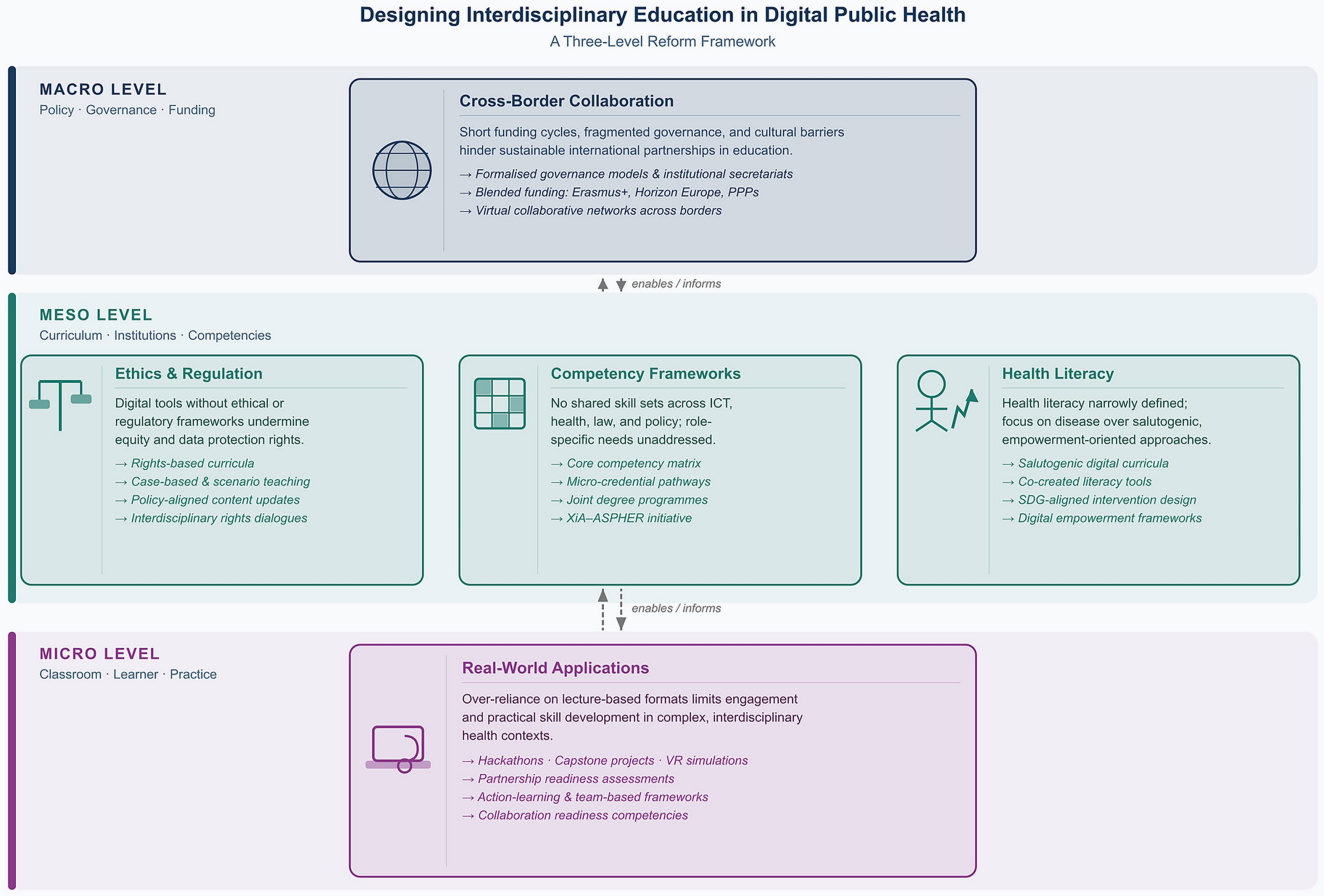
A three-level reform framework for interdisciplinary education in digital public health, comprising the micro level (classroom and learner practice), the meso level (curriculum, institutions, and competencies), and the macro level (policy, governance and funding).

The thematic insights emerging from workshop deliberations on the five key challenges provided the conceptual basis for a three-level framework for interdisciplinary digital public health education, depicted in Figure 1.

**Figure 2:**
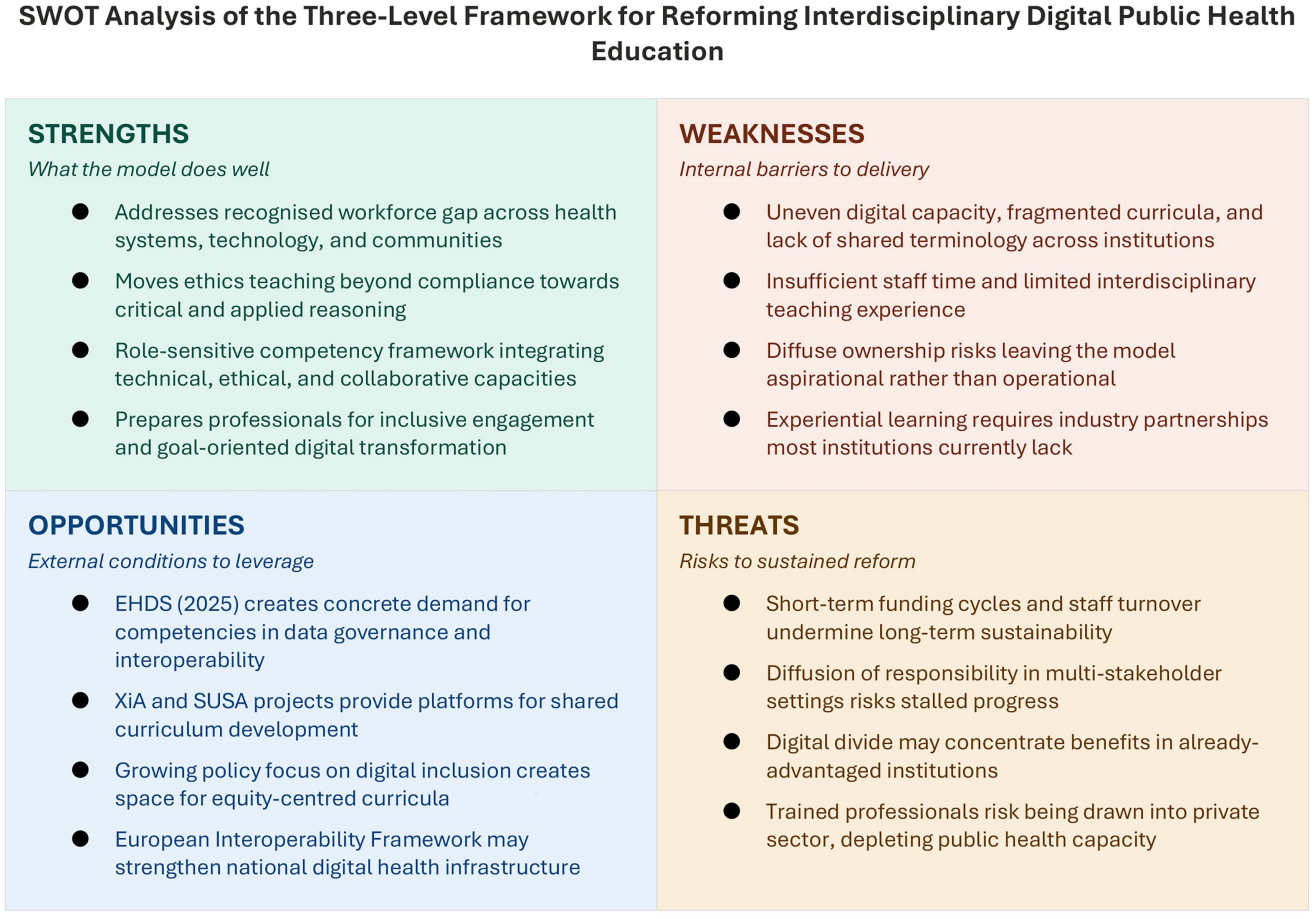
SWOT analysis.

While the framework outlined in Figure 1 provides a structured response to the five challenges identified, its successful implementation is contingent on a range of internal and external factors. Figure 2 presents a SWOT analysis critically appraising the framework’s strengths and limitations alongside the contextual opportunities and threats shaping its implementation. Without explicit accountability mechanisms and diversified, long-term funding commitments, the model risks remaining an aspirational framework rather than an operational reality.

The SWOT analysis highlights several structural risks that warrant particular attention. Weak governance frameworks across participating institutions may hinder coordinated implementation, particularly where no single actor assumes clear ownership of the reform agenda. Sustained funding remains a critical vulnerability, as short-term project cycles and reliance on competitive grants undermine the continuity required for meaningful curriculum change. Staff shortages and high turnover further compound these challenges, given that interdisciplinary teaching demands specialist expertise that is difficult to recruit and retain. Collectively, these risks point to the need for deliberate structural investment, in governance, funding, and accountability, if the framework is to move from aspiration to practice.

## 4 Conclusion

This paper examined five interconnected challenges shaping the future of digital public health education. Embedding ethics, human rights, and data protection into curricula necessitates a shift from compliance-based instruction towards critical and applied reasoning. Strengthening digital and health literacy requires preparing professionals capable of navigating complex digital environments and promoting inclusive engagement. Defining interdisciplinary competency frameworks demands role-sensitive approaches integrating technical, ethical, and collaborative capacities. Advancing experiential learning requires purposeful alignment between pedagogical design and real-world practice. Sustaining cross-border collaboration depends on stable governance structures, long-term partnerships, and diversified funding mechanisms.

Collectively, these priorities underline the need for coordinated reform at institutional, national, and international levels, necessitating the active engagement of educators, health systems, policymakers, students, and communities. Addressed coherently, they represent a significant opportunity to develop a public health workforce equipped to harness digital innovation in advancing health equity and strengthening health systems.

Sustaining such progress, however, requires structural conditions that transcend individual initiatives or funding cycles. Three dimensions are foundational. First, long-term funding stability: current dependence on short-term competitive grants undermines the iterative curriculum reform these challenges demand, necessitating diversified funding streams and sustained budget allocations. Second, institutional commitment: universities and health authorities must formally designate digital public health education as a strategic priority, with dedicated roles, protected staff time, and collaboration embedded in institutional agreements. Third, robust monitoring and evaluation frameworks: without agreed indicators encompassing competency development, learner diversity, partnership stability, and curriculum uptake, demonstrating impact and sustaining investment remains untenable. Together, these three dimensions constitute the structural foundation upon which the five identified challenges may be addressed as a continuous, self-improving educational system.

## Data Availability

The original contributions presented in the study are included in the article/supplementary material, further inquiries can be directed to the corresponding author.

## Footnotes

1
https://xia-project.iscte-iul.pt/
